# Recruitment and Activation of RSK2 by HIV-1 Tat

**DOI:** 10.1371/journal.pone.0000151

**Published:** 2007-01-17

**Authors:** Claudia Hetzer, Dwayne Bisgrove, Michael S. Cohen, Angelika Pedal, Katrin Kaehlcke, Anja Speyerer, Kerstin Bartscherer, Jack Taunton, Melanie Ott

**Affiliations:** 1 Deutsches Krebsforschungszentrum, Heidelberg, Germany; 2 Gladstone Institute of Virology and Immunology, University of California San Francisco, San Francisco, California, United States of America; 3 Department of Cellular and Molecular Pharmacology, University of California San Francisco, San Francisco, California, United States of America; Institut Pasteur, France

## Abstract

The transcriptional activity of the integrated HIV provirus is dependent on the chromatin organization of the viral promoter and the transactivator Tat. Tat recruits the cellular pTEFb complex and interacts with several chromatin-modifying enzymes, including the histone acetyltransferases p300 and PCAF. Here, we examined the interaction of Tat with activation-dependent histone kinases, including the p90 ribosomal S6 kinase 2 (RSK2). Dominant-negative RSK2 and treatment with a small-molecule inhibitor of RSK2 kinase activity inhibited the transcriptional activity of Tat, indicating that RSK2 is important for Tat function. Reconstitution of RSK2 in cells from subjects with a genetic defect in RSK2 expression (Coffin-Lowry syndrome) enhanced Tat transactivation. Tat interacted with RSK2 and activated RSK2 kinase activity in cells. Both properties were lost in a mutant Tat protein (F38A) that is deficient in HIV transactivation. Our data identify a novel reciprocal regulation of Tat and RSK2 function, which might serve to induce early changes in the chromatin organization of the HIV LTR.

## Introduction

The early phase of HIV transcription is characterized by the absence of the viral transactivator Tat and a block in viral transcription elongation [1]. During this phase, basal activity of the viral promoter is controlled by the local chromatin environment, cellular transcription factors that bind to *cis*-acting elements in the viral promoter, and the processivity of the recruited RNA polymerase II (reviewed in [2]). Although inefficient, basal promoter activity is thought to result in a few full-length viral transcripts and synthesis of Tat. Tat dramatically increases the production of full-length transcripts and renders HIV transcription independent of the chromatin environment at the site of integration [3]. Among its functions, Tat recruits the positive transcription elongation factor b (pTEFb) to the TAR element, an RNA stem loop structure at the 5′ termini of nascent HIV transcripts (reviewed in [4]). The arginine-rich motif in Tat (amino acids 49–57) binds to a bulge structure in TAR, while the Tat transactivation domain (amino acids 1–48) associates with the cyclinT1 component in pTEFb [5]. CyclinT1 exists as a complex with cyclin-dependent kinase 9 (CDK9), which hyperphosphorylates the C-terminal domain of RNA polymerase II, increasing its ability to elongate efficiently on the viral template [6], [7].

Various histones modifications occur during transcriptional activation (reviewed in [8]), and some are specifically linked to transcriptional elongation (reviewed in [9], [10]). Histone acetylation and methylation correlate with transcriptional activity of the HIV promoter [11], [12], [13], [14], [15]. Tat interacts with several transcriptional cofactors that have intrinsic histone acetyltransferase (HAT) activity, including Tip60, p300/CBP, PCAF, TAFII250, human GCN5 [16], [17], [18], [19], [20], [21], [22], [23], and is itself acetylated by p300/CBP, PCAF and human GCN5 [21], [24], [25], [26].

Histones can also be phosphorylated in response to several cellular processes. In particular, the rapid and transient mitogen-induced phosphorylation of serine 10 in the tail of histone H3 has been coupled to the transcriptional activation of immediate-early response genes [27]. This phosphorylation event is an early marker in mitogen-induced gene activation and can control subsequent histone acetylation events [28], [29]. However, for some genes histone phosphorylation and acetylation are independent events [30], [31]. Activation-dependent mammalian kinases with intrinsic histone H3 kinase activity include ribosomal S6 protein kinase 2 (RSK2), mitogen- and stress-activated protein kinase 1 (MSK1) and IκB kinase alpha (IKKα) [32], [33], [34], [35].

RSK2 is one of four RSK serine-threonine kinases that are activated through the mitogen-activated protein kinase (MAPK) signal transduction pathway. RSK family members are unusual because they contain two distinct kinase domains that are both catalytically functional. [36] The N-terminal kinase domain phosphorylates downstream targets and is activated through a sequential phosphorylation cascade involving 3-phosphoinositide-dependent protein kinase 1 (PDK1), the C-terminal kinase domain of RSK2 and ERK1/2 (reviewed in [37]). Inactivating mutations and truncations in the RSK2 gene are responsible for Coffin-Lowry syndrome (CLS), which is characterized by severe mental retardation and progressive skeletal deformations [38], [39], [40], [41]. Fibroblasts from CLS patients were found deficient in histone phosphorylation induced by treatment with epidermal growth factor (EGF), indicating that RSK2 is relevant for mitogen-induced histone phosphorylation [32]. However, more recent results show a prominent role of MSK1/2 in mitogen- and stress-induced histone H3 phosphorylation [31], [42]. RSK2 also phosphorylates transcriptional coactivators and transcription factors, including CBP, CREB and c-fos [43], [44], [45], [46]. The transcription factor ATF4 was identified as a preferred substrate for RSK2 and was linked to the skeletal abnormalities in CLS patients [47].

In this study, we examined the role of histone kinases in Tat transactivation. We have observed that Tat increases phosphorylation of histone H3 associated with the HIV promoter. Our findings identify a novel role of Tat as an adaptor and activator of RSK2 in cells and show that RSK2 activity is required for full transactivation of Tat.

## Results

### Histone H3 hyperphosphorylation at the HIV LTR in the presence of Tat

**Figure 1:**
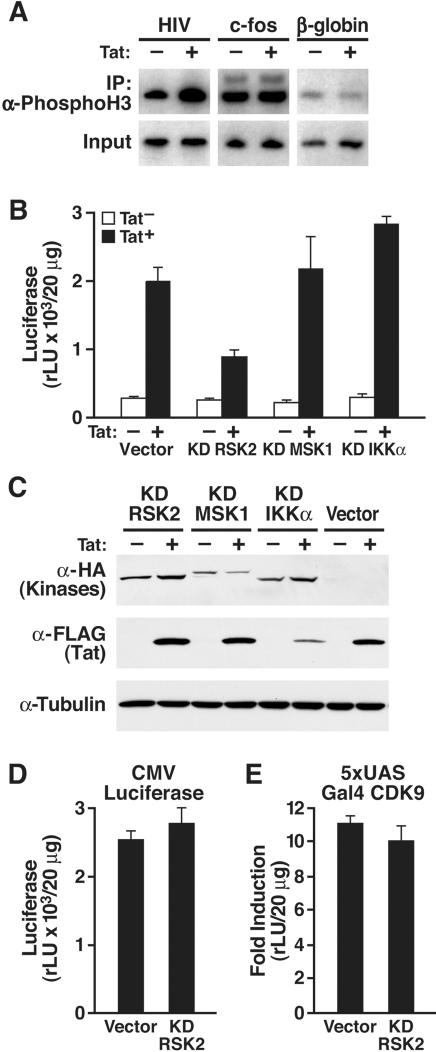
Role of histone kinases in Tat transactivation. (A) Chromatin immunoprecipitation analysis of Jurkat T cells containing an integrated HIV promoter in the absence or presence of Tat. Immunoprecipitations were performed with α-phospho-histone H3 antibodies (serine 10) followed by radioactive PCR with primers specific for the HIV LTR, the c-fos, or the β-globin genes. (B) Jurkat 1G5 cells containing an integrated HIV LTR luciferase construct were transiently transfected with Tat/FLAG (25 ng) and kinase-deficient (KD) kinase expression vectors (200 ng). (C) Western blot analysis of cellular lysates from 293 cells cotransfected with the indicated expression plasmids. (D) Transfection of CMV luciferase (25 ng) with the KD RSK2 expression plasmid (200 ng) in Jurkat cells. (E) Transfection of 5xUAS luciferase and Gal4-CDK9 (20 ng) with the KD RSK2 expression plasmid (200 ng) in Jurkat cells. Values are means±SEM of three experiments.

To study histone H3 phosphorylation of the HIV promoter, we performed chromatin immunoprecipitation (ChIP) studies with antibodies specific for phosphorylated serine 10 in histone H3 (Figure 1A). We used extracts from Jurkat T cells containing an integrated HIV promoter which were either infected with a lentiviral vector expressing the green fluorescent protein (GFP) or Tat [3]. Immunoprecipitations were followed by radioactive PCR with primers specific for the HIV LTR, the c-fos, or the β-globin genes. We observed that HIV LTR-specific sequences were enriched in immunoprecipitates from Tat-expressing cells, indicating that histone H3 phosphorylation at the HIV promoter was increased in the presence of Tat. No difference between GFP- and Tat-expressing cells was detected for the c-fos or β-globin genes, demonstrating that the observed increase was specific for Tat and the HIV promoter.

### Kinase-deficient RSK2 suppresses Tat transactivation

To examine the influence of histone kinases on Tat transactivation, kinase-deficient RSK2, MSK1 or IKKα with known dominant-negative properties were transiently overexpressed in Jurkat 1G5 T cells containing an integrated HIV promoter luciferase construct. Coexpression of kinase-deficient RSK2, but not of kinase-deficient MSK1 or IKKα, inhibited the activity of coexpressed Tat on the HIV promoter (Figure 1B). Basal LTR activity was unaffected when kinase-deficient RSK2 was transfected in the absence of Tat, indicating that the effect of RSK2 is specific for Tat. Expression of kinase-deficient RSK2 had no effect on Tat expression as confirmed by western blot analysis (Figure 1C). Accordingly, kinase-deficient RSK2 did not affect the activity of the cytomegalovirus (CMV) immediate-early promoter that was driving Tat expression in these experiments (Figure 1D). In addition, no effect was observed on the activity of a Gal4-CDK9 fusion protein that transactivates the 5xUAS promoter, excluding the possibility that RSK2 regulates the activity of the Tat-associated kinase pTEFb (Figure 1E).

### RSK2 expression is required for Tat transactivation

**Figure 2:**
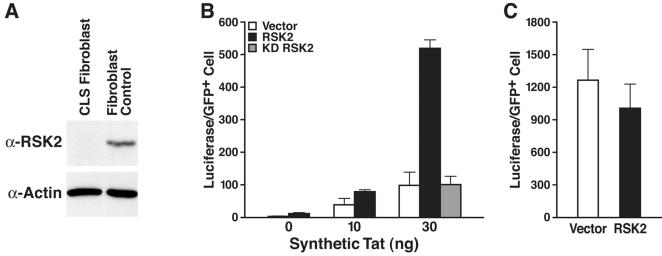
Superinduction of Tat activity in CLS fibroblasts. (A) Western blot analysis of cellular extracts of fibroblasts from a patient with CLS and control human fibroblasts. (B) Nuclear microinjection of CLS fibroblasts with synthetic Tat (amino acids 1–72), the HIV LTR luciferase reporter, a CMV-GFP expression plasmid, and either the empty vector, an RSK2 expression construct, or a plasmid expressing kinase-deficient RSK2. Values are means±SEM of five experiments. (C) Coinjection of the 5xUAS luciferase reporter, a plasmid expressing the Gal4-VP16 transactivator and CMV-GFP with either the RSK2-expressing plasmid or the vector alone. Values are means±SEM of three experiments.

Next, we examined Tat transactivation in a fibroblast cell line from a patient with CLS. No RSK2 protein was detected by western blot analysis (Figure 2A). We used nuclear microinjections to introduce synthetic Tat, the HIV LTR luciferase reporter, and a GFP expression plasmid into these cells. Coinjection of an RSK2-expressing plasmid increased Tat transactivation levels fivefold, demonstrating that RSK2 synergizes with Tat to activate the HIV promoter (Figure 2B). Kinase-deficient RSK2 had no effect, indicating that the catalytic activity of RSK2 is required for synergy with Tat (Figures 2B). No dominant-negative effect of kinase-deficient RSK2 was observed due to the lack of endogenous RSK2 in CLS cells. RSK2 did not affect the 5xUAS promoter activated by the Gal4-VP16 transactivator, indicating that the effect of RSK2 is specific for Tat transactivation (Figure 2C).

### Binding of RSK2 to the transactivation domain in Tat

**Figure 3:**
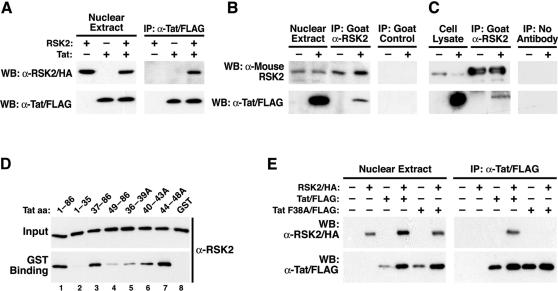
Interaction of Tat and RSK2 *in vivo* and *in vitro.* (A) Coimmunoprecipitation of RSK2 with Tat in nuclear extracts from Cos7 cells cotransfected with RSK2/HA and Tat/FLAG. One representative experiment of five is shown. (B) Coimmunoprecipitation of Tat with endogenous RSK2 in nuclear extracts from Cos7 cells transfected with Tat/FLAG. Immunoprecipitations were performed three times with α-RSK2 or α-CD71 antibodies as a control. (C) Coimmunoprecipitation of Tat with endogenous RSK2 in Jurkat T cells transduced with an HIV-based lentiviral vector expressing Tat/FLAG from the HIV LTR and in uninfected Jurkat T cells. One experiment of two is shown. (D) *In vitro* binding of GST-Tat (amino acids 1–86) or Tat mutants to full-length recombinant RSK2. Western blotting of RSK2 before (Input) and after GST binding is shown. One representative experiment of three is shown. (E) Coimmunoprecipitation of RSK2 with wild type Tat, but not with mutant TatF38A, in nuclear extracts of cotransfected Cos7 cells. Results were confirmed in three independent experiments.

Tat is a nuclear protein, while RSK2 shuttles between the cytoplasm and the nucleus [48], [49]. To determine if the two proteins interact physically in cells, we transfected Cos7 cells with RSK2/HA and Tat/FLAG, and immunoprecipitated the nuclear extracts with α-FLAG agarose. RSK2 coimmunoprecipitated with Tat in cells transfected with RSK2 and Tat expression vectors, but no signal was obtained when RSK2 or Tat was expressed alone (Figure 3A). The same was observed when RSK2 was immunoprecipitated with HA-specific antibodies and recovery of Tat was analyzed (data not shown). Tat also coimmunoprecipitated with endogenous RSK2 in Tat-expressing, but not vector-transfected, Cos7 cells (Figure 3B) and in Jurkat T cells infected with an HIV-based lentiviral vector, where Tat expression was driven by its natural promoter, the HIV LTR (Figure 3C). No RSK2- or Tat-specific signals were obtained after immunoprecipitation with a control antibody (Figure 3B) or with sepharose beads alone (Figure 3C).

To further examine the Tat/RSK2 interaction, recombinant Tat (amino acids 1–86) fused to the C-terminus of glutathione-S-transferase (GST) was incubated with full-length human RSK2 isolated from insect cells. After pulldown with glutathione-sepharose, RSK2 copelleted with GST-Tat 1–86, demonstrating direct interaction between both proteins (Figure 3D, lane 1). RSK2 did not bind to GST, confirming that RSK2 binds the Tat component in the fusion protein (Figure 3D, lane 8). No binding was observed when only the N-terminal 35 amino acids of Tat were included in the reaction, indicating that these residues are not involved in RSK2 binding (Figure 3D, lane 2). Accordingly, binding was not reduced when RSK2 was incubated with a mutant Tat protein lacking amino acids 1–36 (Figure 3D, lane 3). However, deletion of amino acids 1–48 significantly reduced binding to RSK2, indicating that amino acids 37–48 in Tat are involved in RSK2 binding (Figure 3D, lane 4). Amino acids 37–48 correspond to the core region in Tat, which is necessary for Tat transactivation (reviewed in [50]). To confirm that this region is involved in RSK2 binding, we tested binding of RSK2 to three full-length GST-Tat fusion proteins with alanine-scanning mutations in the core region (Figure 3D, lanes 5, 6, 7). Binding to RSK2 was reduced to the greatest extent by mutation of amino acids 36–39, followed by mutations of amino acids 40–43 and amino acids 44–48. Thus, the critical interacting residues lie within amino acids 36–39 in Tat.

Next, we mutated phenylalanine 38 (F38) to alanine within the full-length FLAG-tagged Tat expression vector used in coimmunoprecipitation experiments. After cotransfection of wild type Tat/FLAG or TatF38A/FLAG with RSK2/HA into Cos7 cells, coimmunoprecipitation of RSK2 was only detected for wild type, and not for the mutant, Tat protein (Figure 3E). The difference in detection of RSK2 in the presence of Tat is a variation in RSK2 expression rather than in RSK2 translocation, since the same pattern of RSK2 detection was observed in cytoplasmic extracts from the same experiment (not shown). The variation of Tat expression is unique to this experiment (Figure 3E).

### Transcriptional synergy with RSK2 is lost in TatF38A

**Figure 4:**
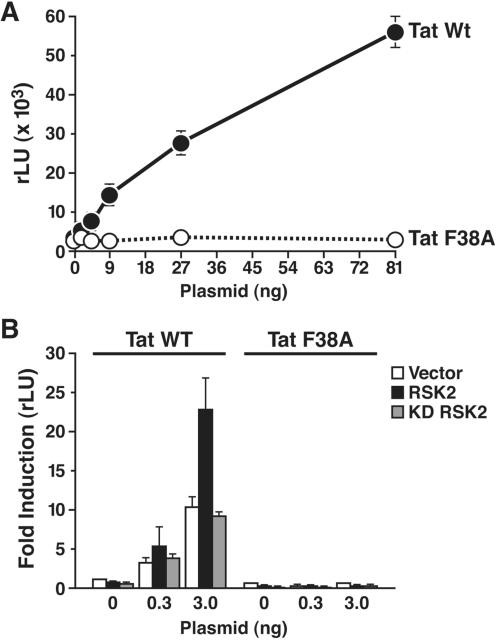
Transcriptional activity of TatF38A. (A) Transfection of indicated amounts of Tat- or TatF38A-expressing constructs in Jurkat 1G5 cells using the DEAE-dextran method. (B) Cotransfection of indicated amounts of Tat or TatF38A with the HIV LTR luciferase (200 ng) and wild type or kinase-deficient RSK2 (200 ng) in CLS cells. Values are means±SEM of five experiments for wild type and three experiments for mutant Tat.

F38 is a highly conserved residue in Tat, and alanine substitution was shown to abrogate Tat transcriptional activity [51]. Others reported normal transactivation of a TatF38 mutant (TatF38L) in transfection assays, but impaired viral growth when the mutation was introduced into infectious HIV [52], [53]. We transfected wild type Tat or TatF38A into Jurkat 1G5 cells and found that the mutation abrogated Tat transactivation of the integrated HIV promoter (Figure 4A). To test whether the mutation affected synergy with RSK2, we cotransfected Tat- and RSK2-expressing constructs together with the HIV LTR luciferase reporter into CLS cells. While RSK2 synergized with wild type Tat, no synergy was observed with TatF38A, supporting the model that RSK2 suppports Tat transactivation via binding to F38 (Figure 4B). Again, kinase-deficient RSK2 had no effect, confirming that RSK2 catalytic activity is required for synergy with Tat (Figure 4B).

### Activation of the N-terminal RSK2 kinase activity by Tat

**Figure 5:**
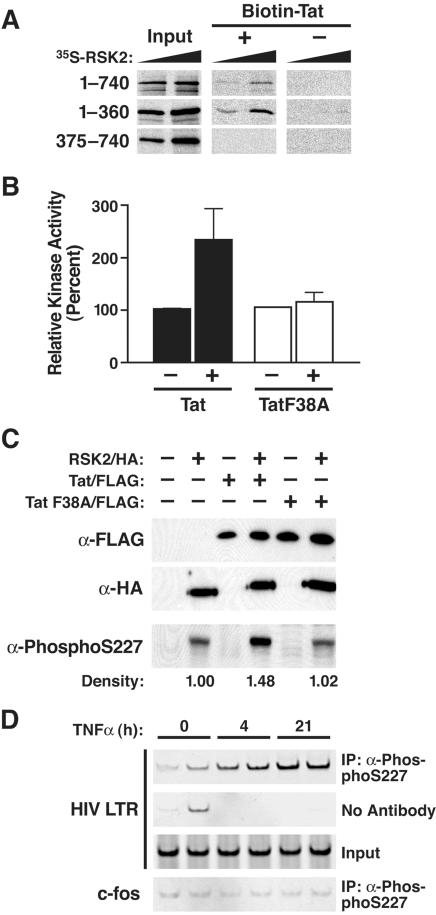
Activation of RSK2 by Tat. (A) Autoradiography of radioactive *in vitro* synthesized RSK2 proteins before (Input) and after binding to biotinylated synthetic Tat (amino acids 1–72) or to beads alone. Increasing amounts of *in vitro* translated RSK2 were included in the binding reaction. (B) Kinase assay of endogenous RSK2 immunoprecipitated from Cos7 cells transfected with wild type Tat/FLAG, TatF38A/FLAG, or empty vector. Values are means±SEM of four experiments. (C) Western blotting of nuclear extracts isolated from Cos7 cells cotransfected with RSK2/HA and Tat/FLAG or with RSK2/HA and Tat F38A/FLAG constructs. Densitometric quantification of the phospho-S227-specific bands was performed using the NIH Image software. (D) Chromatin immunoprecipitation analysis of the Jurkat T cell line A2, latently infected with an HIV-based lentiviral vector expressing Tat/FLAG from the HIV LTR after treatment with TNF-α. At indicated time points, cells were harvested and immunoprecipitations were performed in duplicate with α-phospho-S227 antibodies followed by PCR with primers specific for the HIV LTR or the c-fos gene.

To examine the Tat-interacting domain in RSK2, we performed binding studies using biotinylated synthetic Tat protein (amino acids 1–72) and *in vitro* synthesized radioactive RSK2. We also tested two RSK2 deletion mutants spanning amino acids 1–360 and 375–740, respectively. After pull-down with streptavidin-agarose, full-length RSK2 and RSK2 1–360 interacted in a dose-dependent manner with Tat, indicating that the Tat-interacting domain lies in the N-terminus of RSK2 (Figure 5A). No signal was detected when RSK2 was incubated with streptavidin-agarose alone, excluding unspecific binding in this assay.

Amino acids 1–360 harbor the N-terminal kinase domain, which mediates substrate phosphorylation by RSK2. To determine if Tat binding affects the kinase activity of RSK2, we transfected Cos7 cells with Tat- or TatF38A-expressing constructs and performed standard S6 peptide phosphorylation assays after immunoprecipitation of endogenous RSK2. Expression of wild type, but not mutant Tat increased the kinase activity of endogenous RSK2 threefold (Figure 5B), as did epidermal growth factor (EGF), a natural stimulus of RSK2 kinase activity (not shown).

Activation of RSK2 by Tat was further examined by western blot analysis. After mitogenic stimulation, serine 227 (S227) in RSK2 is phosphorylated, an event that correlates with substrate phosphorylation by RSK2 [54]. We cotransfected RSK2/HA- and Tat/FLAG-expressing constructs in Cos7 cells and examined cell lysates with antibodies specific for phospho-S227 in RSK2. Phosphorylation in RSK2 was enhanced by wild type Tat, but not TatF38A, indicating that Tat binding to RSK2 is required for the increase in kinase activity and S227 phosphorylation of RSK2 (Figure 5C). Reprobing of the membrane with HA- and FLAG-specific antibodies showed similar levels of RSK2/HA and both Tat proteins throughout the experiment (Figure 5C).

To test whether Tat recruits RSK2 to the HIV promoter, we performed ChIP assays with α-phospho-S227 antibodies. Chromatin solutions were extracted from Jurkat T cells containing a single integrated latent HIV genome. Following TNF-α treatment, HIV transcription is activated in these cells and Tat is produced. The DNA immunoprecipitated along with phospho-S227 was analyzed by PCR with primers specific for the HIV promoter or for the cellular *c-fos* gene. After induction with TNF-α, the HIV LTR-specific sequence was enriched in the immunoprecipitated fraction, demonstrating that phospho-RSK2 is recruited to the HIV LTR *in vivo* (Figure 5D). TNF-α treatment alone did not activate S227 phosphorylation in the absence of Tat, suggesting that the recruitment or the activation of RSK2 at the HIV promoter occurred specifically in the presence of Tat (data not shown). No enrichment was observed for the *c-fos*-specific sequence or when α-phospho-S227 antibodies were omitted from the reaction (Figure 5D).

### Suppression of HIV transcription by FMK, a novel inhibitor of RSK2

**Figure 6:**
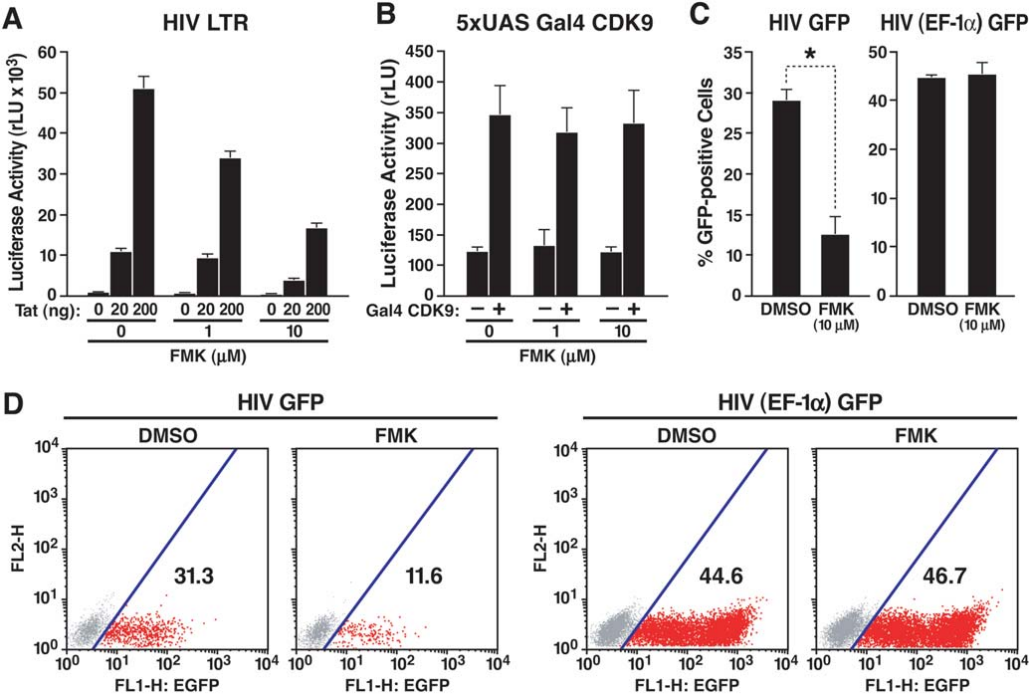
Suppression of HIV transcription by FMK, a small-molecule inhibitor of RSK2. (A) Transient transfection of Jurkat 1G5 cells, containing an integrated HIV LTR luciferase construct, with Tat/FLAG (20 and 200 ng). Transfected cells were treated with indicated amounts of FMK or DMSO for 18 h. Values are mean±SEM of four experiments. (B) Cotransfection of Jurkat T cells with 5xUAS luciferase and Gal4-CDK9 (20 ng) and subsequent treatment with FMK at indicated concentrations. Values are means±SEM of four experiments. (C) GFP expression in Jurkat T cells infected with HIV_NL4-3_ containing the GFP open reading frame in place of the viral *nef* gene or with an HIV-based lentiviral vector expressing GFP from the heterologous EF-1α promoter. After overnight infection, cells were treated with FMK or DMSO for 36 h. Values are means±SEM of three experiments. *p = 0.002 (*t* test). (D) GFP expression in one representative experiment performed with HIV GFP or HIV (EF-1α) GFP virus.

Recently, a structural bioinformatics-based approach was used to design selective inhibitors of RSK1, 2, and 4, including the compound FMK, which at 1 or 10 µM inactivates RSK1 and RSK2 in mammalian cells [55]. Treatment of Jurkat 1G5 cells with FMK suppressed Tat-dependent HIV transcription in a dose-dependent manner after transfection of the Tat vector (Figure 6A). Importantly, FMK did not suppress the transcriptional activity of the Gal4-CDK9 fusion protein, confirming that the effects of RSK2 on Tat transactivation are not mediated by pTEFb (Figure 6B).

To examine the effect of FMK on HIV infection, we generated infectious HIV particles using a molecular clone of HIV_NL4-3_ that contains the GFP open reading frame in place of the viral *nef* gene [56]. This clone also contains a frameshift mutation in the viral *env* gene, restricting analysis to a single infection cycle. Viral particles were pseudotyped with VSV-G. Jurkat T cells were incubated with viral supernatant, washed, and treated with FMK (10 µM) or DMSO alone. FMK reduced HIV gene expression two- to threefold, as measured by GFP expression, but had no effect on GFP expression in cells infected with an HIV-based lentiviral vector expressing GFP from the elongation factor 1α (EF-1α) promoter (Figures 6C and D). These data indicate that the RSK2 kinase activity regulates viral gene expression during HIV infection.

## Discussion

This study shows that RSK2 is a novel regulator of Tat transactivation, a conclusion supported by three independent observations. First, a dominant-negative form of RSK2, but not of other histone kinases, inhibited Tat transactivation. This effect was specific for Tat since no effect was observed on basal promoter activity of the HIV LTR or on the Tat-independent CMV promoter. Second, cells from CLS patients, which lack RSK2, exhibited a transcriptional defect. Ectopic expression of RSK2, but not of a catalytically inactive mutant, enhanced Tat transactivation in CLS cells, demonstrating that the kinase activity of RSK2 is important for Tat transactivation. In contrast, RSK2 overexpression in CLS cells slightly inhibited the transcriptional activity of the VP16 transactivator, demonstrating that RSK2 targets Tat specifically and that its overexpression does not induce general cell hyperactivation. Third, the RSK inhibitor FMK suppressed Tat transactivation and HIV gene expression. This effect was specific for HIV transcription, since no effect was observed on the infection with control viral vectors, in which GFP expression was driven by the cellular EF-1α promoter.

A Tat mutant (F38A) that does not bind RSK2 exhibited a transcriptional defect that cannot be attributed exclusively to RSK2. F38 lies in the core domain of Tat, which is involved in binding of other transcriptional cofactors, including cyclinT1 and PCAF [5], [22], [23]. Interestingly, kinase-deficient RSK2 and the RSK inhibitor FMK had no effect on the transcriptional activity of CDK9, the binding partner of cyclinT1 and the kinase component of pTEFb. This finding excludes the possibility that RSK2 regulates Tat transactivation through pTEFb.

The observation that Tat activates the kinase activity of RSK2 is unexpected and shows an important novel function of Tat. The enzymatic activity of RSK2 is regulated by sequential phosphorylation events. Phosphorylation by ERK1/2 displaces an inhibitory α-helix from the substrate-binding region of the C-terminal kinase domain of RSK2 and activates its kinase activity [57], [58]. This leads to autophosphorylation of serine 386 (S386) in the linker region between the kinase domains, which recruits and activates PDK1 [59]. PDK1 phosphorylates S227 in the N-terminal kinase domain, the final step in the phosphorylation cascade, and induces substrate phosphorylation.

We have shown that this final step is activated by Tat. It is not clear whether Tat binding leads to immediate phosphorylation of S227 or involves the classical MAPK-induced activation cascade. Interestingly, ERK1 expression might be decreased in cells expressing Tat [60]. Direct activation of RSK2 by Tat, independently of upstream activation signals, could therefore compensate for a decrease in ERK1 expression and signaling during HIV infection. We and others observed that U0126, an inhibitor of MAPK/ERK kinase (MEK), the kinase upstream of ERK, had no effect on Tat transactivation, supporting the notion that Tat targets RSK2 directly (data not shown; [61]). Moreover, FMK, a direct inhibitor of RSK2 kinase activity, suppressed Tat transactivation in a dose-dependent manner. FMK selectively inhibits the C-terminal kinase domain and targets a reactive cysteine that, together with a threonine “gatekeeper,” controls access of FMK to the ATP binding pocket [55]. A recent study found MAP kinase p38α activated through direct binding by the adaptor protein transforming growth factor-β-activated protein kinase 1-binding protein 1 (TAB1) in the absence of upstream signals [62]. It was proposed that TAB1 binding induced a conformational change in p38α, which activated its kinase activity and enhanced the level of kinase autophosphorylation. One possibility is that Tat through binding to the N-terminal kinase domain, which does not exhibit substantial intrinsic autophosphorylation activity [58], alters the structural conformation of RSK2 and stimulates the autophosphorylation activity of the C-terminal kinase domain, leading to phosphorylation of S386 and subsequently S227. In addition, by direct binding to the N-terminal kinase domain, Tat could activate S227 phosphorylation through an unknown mechanism.

Our experiments show that phosphorylation of histone H3 is enhanced in Tat-expressing T cells. In addition, we show that activated RSK2 is recruited to the HIV promoter *in vivo*, where it can target histones. We and others have previously shown that Tat also recruits the HAT activity of PCAF via its acetylated lysine 50 [14], [22], [23], [63]. Since at some promoters acetylation of lysine 14 in histone H3 – mediated by PCAF - is enhanced when serine 10 is phosphorylated, the two functions of Tat can be expected to synergize in the stimulation of HIV transcription. However, in our experiments the positive effect of RSK2 on Tat transactivation was observed on integrated as well as nonintegrated LTR promoter templates, which may indicate that RSK2 targets additional substrates besides histones. Future experiments will address the role of RSK2 in the phosphorylation of histone H3 or other known RSK2 substrates at the HIV promoter, including CBP, CREB, c-fos and ATF4.

## Material and Methods

### Cells and plasmids

Cos7, HeLa, 293, and Jurkat cells (American Type Culture Collection), Jurkat 1G5 cells (AIDS Research and Reference Reagent Program), and CLS fibroblasts (Coriell Institute for Medical Research, Camden, NJ) were cultured under standard cell culture conditions. Jurkat 1G5 cells were transiently transfected with the DEAE-dextrane technique, and luciferase activity was measured 20 h later (Promega).

Jurkat cells were stably infected with lentiviral vectors expressing Tat/FLAG [56], [64]. Constructs encoding N-terminal HA-tagged wild type and dominant-negative RSK2 (K100A) were provided by M. Greenberg (Harvard Medical School, Boston, Massachusetts, USA) [45], dominant-negative IKKα (K44M) by W. Greene and A. O'Mahony (Gladstone Institute of Virology and Immunology, San Francisco, California, USA) [65], dominant-negative MSK1 (R102A) and HA-tagged RSK2 and deletion mutants by M. Frodin (Department of Clinical Biochemistry, Glostrup Hospital, 2600 Glostrup, Denmark) [59], [66]. NotI/XhoI fragments from these RSK2 vectors were blunted and subcloned into the EcoRV site of pcDNA3.1 (Invitrogen) for *in vitro* transcription/translation (Promega). GST-Tat 1–86 and 1–48 were obtained from the AIDS Research and Reference Reagent Program. All other GST-Tat deletion constructs were a gift from Q. Zhou and D. Chen (Department of Molecular and Cell Biology, University of California, California, USA) [67]. The 5xUAS construct, in which 5 x Gal4 binding sites were cloned upstream of the TK promoter [68], was provided by B. Spiegelman (Dana-Farber Cancer Institute and Department of Cell Biology, Harvard Medical School, Boston, Massachusetts, USA) and the Gal4-CDK9 construct by M. Peterlin (Department of Medicine, Rosalind Russell Medical Research Center, University of California, San Francisco, California,USA) [69]. Full-length Tat (101 amino acids) bearing a C-terminal FLAG tag [25] and the LTR luciferase construct [70] have been described. The Tat-FLAG construct served as template for site-directed mutagenesis (Stratagene) to generate the F38A mutation. The CMV luciferase construct was generated by cloning the luciferase gene as a HindIII/BamHI fragment obtained from pGL2 Basic (Promega) into pcDNA3.1 (Invitrogen). The CMV-GFP construct was purchased from Clontech.

### ChIP Assays

Jurkat cells, 24 h after transduction with lentiviral vectors encoding either Tat or GFP and Jurkat clone A2 containing a single integrated latent HIV minigenome induced with TNF-α (10 ng/ml; Biosource) were fixed with 1% formaldehyde, partially digested with micrococcal nuclease (Roche) and sonicated as described [71]. Precleared chromatin solutions were incubated overnight at 4°C with 1 µg of rabbit α-phospho-histone H3 (S10; Upstate Biotechnology) or 5 µg of rabbit α-phospho-S227 (Santa Cruz Biotechnology). Immune complexes were collected with protein A-sepharose preblocked with sonicated salmon sperm DNA (Upstate Biotechnology). Formaldehyde cross-links were reverted by incubating the samples at 65°C overnight in the presence of 200 mM NaCl. One-tenth of the immunoprecipitated DNA was used in PCR reactions using primers previously described [64].

### Viral infection experiments

Jurkat T cells infected with lentiviral vectors expressing Tat/FLAG or GFP used in coimmunoprecipitation experiments have been described [64]. For studies with the RSK2 inhibitor FMK, Jurkat cells were infected with a molecular clone of HIV containing the GFP open reading frame in place of *nef* [56]. FMK is a pyrrolopyrimidine that contains a fluoromethylketone. Minimal lentiviral vectors driving GFP expression from a heterologous promoter (EF-1α) served as a control (pHR'-EF-1α/GFP) [72]. Viral particles were pseudotyped with vesicular stomatitis virus glycoprotein (VSV-G). All vectors and protocols to generate lentiviral particles were provided by D. Trono (EPFL SV-DO, Lausanne, Switzerland). Jurkat T cells were incubated overnight with lentiviral particles at a theoretical multiplicity of infection (m.o.i.) of 0.5 in 24-well plates. Cells were repeatedly washed and resuspended in fresh medium containing FMK (1 or 10 µM) or DMSO alone. Viral infection was monitored 36 h later by flow cytometric analysis using a Calibur FACScan (Beckton Dickinson).

### Nuclear microinjections and transfections of CLS cells

Subconfluent CLS fibroblasts were grown on Cellocate coverslips (Eppendorf) and were microinjected at room temperature with an automated injection system (Carl Zeiss) or a micromanipulator/transjector (Eppendorf). The medium was buffered during the microinjection by adding 20 mM HEPES buffer. Samples were prepared as a 20 µl injection mix containing synthetic Tat (30 ng/µl) [23], [64] and the LTR luciferase (100 ng/µl) and CMV-GFP (50 ng/µl) constructs together with the CMV-RSK2 expression construct or the empty vector (each 200 ng/µl). Live cells were examined on a Zeiss Axiovert or a Nikon Eclipse TE300 microscope to determine the number of GFP-positive cells. Four hours after injection, cells were washed in cold PBS and processed for luciferase assays (Promega).

For transfections, subconfluent CLS fibroblasts in six-well plates were transfected using Lipofectamine reagent (Invitrogen) and Tat-expressing plasmid (0.3 and 3 ng), HIV LTR luciferase reporter (200 ng) and RSK2-expressing constructs or empty vector (each 200 ng). Cells were harvested twenty-four hours after transfections and equal amounts of cellular extract (as measured by protein content) processed for luciferase assays (Promega).

### Coimmunoprecipitation and western blot analysis

Cos7 cells (3×10^5^) were transfected with Tat- and RSK2-expressing plasmids (6 µg) with Lipofectamine reagent (Invitrogen). Twenty-four hours after transfection, nuclear extracts were prepared in hypotonic buffer (HB) as described [43]. Equal amounts of nuclear extracts were precleared with protein A- or G-sepharose (Amersham Pharmacia Biotech) that had been blocked with 50 mg/ml BSA in HB for 1 h at 4°C. Precleared nuclear extracts were immunoprecipitated with α-FLAG (Sigma), α-HA (Roche), or α-RSK2 (Santa Cruz Biotechnology) antibodies and protein A- or G-sepharose overnight at 4°C in HB supplemented with 120 mM NaCl. Pellets were washed five times in HB supplemented with 120 mM NaCl, resuspended in Laemmli buffer, and analyzed by western blot with α-FLAG, α-HA or α-RSK2 antibodies.

Tat and endogenous RSK2 were coimmunoprecipitated from cellular extracts isolated from Jurkat cells or Jurkat cells infected with a lentiviral vector expressing Tat/FLAG under the control of the HIV LTR [64]. Western blot analysis with phospho-S227-specific antibodies (Santa Cruz Biotechnology) was performed as described [59].

### Kinase assays

Nuclear extracts from Cos7 cells transfected with Tat/FLAG, Tat F38A/FLAG, or vector alone were immunoprecipitated with α-RSK2 antibodies as described above. Immunoprecipitates were washed three times in HB supplemented with 120 mM NaCl and once in kinase assay buffer [43]. Kinase assays were performed with a standard S6 kinase protocol (Upstate Biotechnology).

### *In vitro* binding assays

Constructs encoding wild type RSK2 or deletion mutants were subjected to *in vitro* transcription/translation in the presence of 20 µCi [^35^S]-methionine (Amersham Pharmacia Biotech) using the TNT T7-coupled reticulocyte lysate system (Promega). Biotinylated synthetic Tat protein [23], [64], immobilized to streptavidin-sepharose, was incubated with 0.5 or 1.5 µl of reticulocyte lysate containing radioactive RSK2 proteins in HB buffer supplemented with 120 mM NaCl described above. Binding reactions were incubated for 10 min at 30°C, washed three times in binding buffer containing 1 M KCl, and analyzed by SDS-PAGE. The gel was fixed, amplified (Amersham Pharmacia Biotech), and exposed to BioMax MR film (Kodak).

GST fusion proteins were expressed in the BL21 strain of *Escherichia coli* and purified with glutathione-sepharose as described [6]. Bound proteins were eluted with 25 mM glutathione (Sigma), dialyzed (50 mM Tris, pH 8.0, 20 mM NaCl, 0.5% NP-40, 5 mM DTT, 50 µg/ml AEBSF, 15% glycerol), and protein concentrations were measured with the RC DC protein assay (Bio-Rad) and verified after SDS-PAGE by Coomassie staining or western blot analysis.

For *in vitro* binding studies, equal amounts of GST proteins (1 µg/µl) were immobilized to glutathione-sepharose beads that had been blocked with 50 mg/ml BSA in binding buffer (20 mM Hepes, pH 7.9, 1% Triton, 0.5% NP-40, 200 mM KCl, 0.1% BSA, 2 mM DTT, protease inhibitor cocktail). After three washes in EBC/DTT/SDS (50 mM Tris, pH 8.0, 20 mM NaCl, 0.5% NP-40, 5 mM DTT, 50 µg/ml AEBSF, 0.075% SDS, protease inhibitor cocktail) and one wash in binding buffer, the beads were incubated with 200 ng of human recombinant RSK2 (Upstate Biotechnology). Bead-coupled proteins were washed three times in binding buffer containing 1 M KCl and boiled in Laemmli buffer, and supernatants were analyzed by western blot analysis with α-RSK2 antibodies (Santa Cruz Biotechnology).
